# Assessing Differences in Measurement of Nonfatal Firearm Crime in the Uniform Crime Report Summary Reporting System and National Incident-Based Reporting System

**DOI:** 10.1007/s11524-026-01109-8

**Published:** 2026-07-01

**Authors:** Nicholas S. Meyerson, Cassandra K. Crifasi, Mo Li, Ni Zhao, Daniel W. Webster

**Affiliations:** 1 Center for Gun Violence Solutions, Department of Health Policy and Management, Johns Hopkins Bloomberg School of Public Health, Baltimore, MD USA; 2 Department of Mathematics, University of Louisiana at Lafayette, Lafayette, LA USA; 3 Department of Biostatistics, Johns Hopkins Bloomberg School of Public Health, Baltimore, MD USA

**Keywords:** Firearm violence, Nonfatal shooting, Crime reporting

## Abstract

Data collection processes of fatal firearm violence are well established. These processes are far more fragmented for nonfatal firearm violence—specifically injuries sustained in the commission of a crime. Until recently, no national databases systemically tracked nonfatal gunshot wounds. In this study, we aimed to enhance understanding of this subject by assessing concordance between measures of nonfatal firearm crimes from two national data sources: the Federal Bureau of Investigation’s Uniform Crime Report Summary Reporting System (UCR-SRS) and the National Incident-Based Reporting System (NIBRS). We obtained data from 85 law enforcement agencies that served large jurisdictions and reported data to the UCR-SRS and NIBRS for at least 10 years between 2000 and 2020. We calculated annual ratios and cross-sectional and temporal correlations between NIBRS incidents with a firearm resulting in an injury and UCR firearm-related crimes for aggravated assaults and robberies. We performed a hierarchical cluster analysis to identify agencies with similar clusters of correlations between the two measures. We found that approximately 87% of UCR-reported incidents with a firearm had no reported victim that experienced a likely gunshot injury. Among agencies, 60% exhibited low positive to negative correlations between NIBRS and UCR for at least one of the two crime categories. Our findings show the difficulty in estimating the incidence and trends of nonfatal firearm violence with crime data. Advancements made by NIBRS are critical to collect more comprehensive data on nonfatal firearm violence, but gaps in participation and discrepancies in reporting across agencies must be addressed to realize the data system’s potential. Keywords: Firearm violence, Nonfatal shooting, Crime reporting

## Introduction

Between 2019 and 2021, the United States (U.S.) experienced an unprecedented increase in firearm violence. Over this period, the firearm homicide rate increased by 45% compared with a 7% increase in non-firearm homicides [1, 2]. Since this spike, the rate of firearm homicides has declined markedly, from 6.6 per 100,000 in 2021 to 4.7 per 100,000 in 2024 [2]. Despite communities reporting widespread reductions in firearm homicides and shootings [3], public safety and crime remain a significant concern among Americans [4]. Data on firearm homicides are well documented, but the full extent of individuals shot in the commission of a crime each year has remained unknown because of inadequacies in the current data surveillance system.

For many decades, U.S. jurisdictions had no standard system for collecting data on individuals who experienced nonfatal gunshot injuries from criminal acts of violence. Prior literature has leveraged survey data (i.e., National Crime Victimization Survey), open-source data (i.e., The Gun Violence Archive), and public health and healthcare databases (i.e., the Center for Disease Control and Prevention’s Web-based Injury Statistics Query and Reporting System and the Healthcare Cost and Utilization Project) [5, 6]. However, these different data sources suffer from issues of reliability, representativeness, and generalizability [5, 7]. For instance, healthcare databases rely on hospital medical records, which often misclassify intentional injuries as accidents [8]. Some studies have narrowed their focus to interpersonal violence and have relied on data derived from criminal-legal sources [7, 9, 10]. Specifically, the Uniform Crime Reporting Program’s Summary Reporting System (UCR-SRS), published by the Federal Bureau of Investigation (FBI), reports monthly counts of robberies and aggravated assaults committed with specific categories of weapons, including firearms. However, UCR-SRS reporting on firearm-involved incidents does not include data on victim injuries that would allow data users to distinguish between cases in which victims sustained nonfatal gunshot wounds, those in which victims were shot at but not hit, and those in which a firearm was used to threaten but was not fired [11]. Additionally, the UCR-SRS instituted a “hierarchy rule” in which only the most serious crime that occurred during an event (based on a predefined order of offenses by severity) was recorded [11]. Hence, if a robbery and an aggravated assault occurred during the same incident, only the robbery would be reported to the UCR program because it ranks higher on the hierarchy.

On January 1, 2021, the FBI transitioned from using the UCR-SRS to using only the National Incident-Based Reporting System (NIBRS). Prior to that change, the FBI encouraged agencies to report to the NIBRS to enhance the quality of national crime statistics by capturing more detailed information on incident-level characteristics. However, incomplete reporting of crime data in 2021 led the FBI to continue accepting data through UCR-SRS while encouraging NIBRS participation. In 2023, 83% of the U.S. population was covered by NIBRS reporting [12, 13]. Researchers continue to grapple with how to compare crime reported through the two systems. In a prior comparison, Parker concluded that the UCR-SRS and NIBRS did not differ in the proportion of violent crimes that involved a firearm, but the UCR-SRS underestimated the number of firearms crimes by approximately 65,000 events annually [14]. Although the national transition to NIBRS should improve the quality of data collected [15], it is critical for researchers to understand how to use and interpret historic crime data appropriately [7].

The broad heterogeneity within FBI crime categories can potentially cloud assessment of nonfatal firearm violence. For instance, a nonfatal criminal incident could involve a firearm that was brandished, aimed at someone, fired but without wounding any victim, or fired resulting in any number of victims with life-threatening gunshot wounds. Across and within these examples of firearm crimes, there are likely to be differences in the probability the incident will be reported to police [16]. Victims of firearm violence may not wish to report the incident to police due to their own criminal involvement or due to mistrust of law enforcement [17, 18]. Additionally, gun crimes in which a victim sustains a nonfatal gunshot injury may differ in important ways from gun crimes in which there are no gunshot victims. As many studies of firearm violence and efforts to prevent it are based on comparisons over time and across jurisdictions, there are implicit assumptions that the reporting of firearms crimes is consistent across time and place. Thus, this study is intended to examine how closely UCR-SRS measures of nonfatal gun crimes mirror patterns and trends in nonfatal firearm crimes resulting in injuries consistent with gunshot wounds using data from NIBRS. The comparison between UCR-SRS and NIBRS also allows us to investigate differences in the reporting of nonfatal firearm crimes without any victim who sustained injuries across time and place.

Despite increased awareness and acknowledgement of firearm violence as a public health crisis, public surveillance and data collection of nonfatal firearm injuries remain inadequate. In this study, our goal was to assess how well UCR measures of nonfatal firearm crimes—aggravated assaults and robberies with a firearm—reflect differences over time and place in a measure of nonfatal firearm crimes resulting in injuries consistent with gunshot wounds reported to NIBRS.

## Methods

We obtained victimization and offense data from the UCR-SRS and NIBRS between 2000 and 2020. We limited our sample to law enforcement agencies that served populations of 100,000 or more during at least 1 year of the study period and reported data concurrently to UCR-SRS and NIBRS for at least 10 years of the study period. To ensure completeness of data, we only considered years in which an agency reported data for all 12 months (an agency-year) to both UCR-SRS and NIBRS. We identified 85 agencies that met the inclusion criteria.

To ascertain annual counts of NFS from NIBRS, we separately aggregated the number of aggravated assaults and robberies committed with a firearm where a victim was injured but did not die. The proxies we used for a nonfatal gunshot wound were based on NIBRS injury codes and included “possible internal injury,” “severe laceration,” or other major injury. We excluded NIBRS injury codes for apparent broken bones, apparent minor injury, loss of teeth, or unconsciousness that could result from blunt-force trauma. Although the system recorded victim injuries, “gunshot wound” was not listed as an option in NIBRS. In June 2025, for the first time, the NIBRS provided a mechanism for law enforcement agencies to document nonfatal gunshot wounds. Among the 85 identified agencies, we then collected data from the UCR on aggravated assaults and robberies with a firearm. We aggregated annual counts for each category indexed by agencies.

We ran quality tests to determine if injury and weapon codes were used in data reporting. When an agency reported overall crime categories but did not report the use of weapons (UCR-SRS and NIBRS) or occurrence of injuries (NIBRS), we omitted the agency-year observation from subsequent analyses (*n* = 23 agency-years).

We compared annual ratios among agencies between NIBRS incidents with a firearm resulting in a nonfatal injury (NIBRS NFS) and UCR firearm-related crimes (UCR gun crimes) for assaults and robberies. We then calculated cross-sectional and temporal correlations between counts of NIBRS NFS and UCR gun crimes for assaults, robberies, and across crime categories. To assess the degree and frequency of discordance between changes in the ratio of NIBRS and UCR measures over time and across agencies, we used Spearman’s rank correlations. We compared year-to-year differences in the ratio of NIBRS NFS to UCR gun crimes for each agency. Then, with the calculated Spearman’s rank correlations, we performed a hierarchical cluster analysis using the complete linkage method and Euclidean distance metrics to identify cities with similar clusters of correlations between NIBRS NFS and UCR gun crimes. We used a Circos plot to visually represent the correlation patterns across all 85 agencies, facilitating the division of the clustered tree structure and illustrating the identified groups.

## Results

The 85 identified agencies included a total of 1785 agency-year observations (Table 1). More than 98% of agency-year observations had complete data from the UCR-SRS (*n* = 1754) and approximately 87% of observations had complete data from NIBRS (*n* = 1545). In instances of missing data for the UCR-SRS (*n* = 31 agency-years), 67.7% reported incomplete data, 16.1% had no reported data, and 16.1% were excluded because of suspected data entry error in crime categories. For the NIBRS, most agency-years with missing data (*n* = 240) resulted from no data being reported (85.8%). The remaining cases either had incomplete data for the full year (9.6%) or reported overall crime categories but did not systematically report injuries sustained (2.5%). We excluded five agency-year observations (2.1%) because of suspected data entry error in crime categories based on yearly trends. Our final analytic sample included agency-year observations with complete UCR-SRS and NIBRS data (*n* = 1543).

**Table 1:** Summary statistics of reporting systems

	UCR-SRS			NIBRS		
	Average (*n*)	Median (*n*)	SD	Average (*n*)	Median (*n*)	SD
Years reported	20.7	21	1.11	18.8	21	3.02
**Gun crimes**						
Aggravated assault with firearm	330.03	90	711.64	305.47	98	644.95
Robbery with firearm	249.16	67	520.68	315.35	104	627.18
**Nonfatal shootings**						
Aggravated assault with firearm and injury	-	-	-	45.52	18	82.61
Robbery with firearm and injury	-	-	-	39.13	10	81.24

**Fig. 1:**
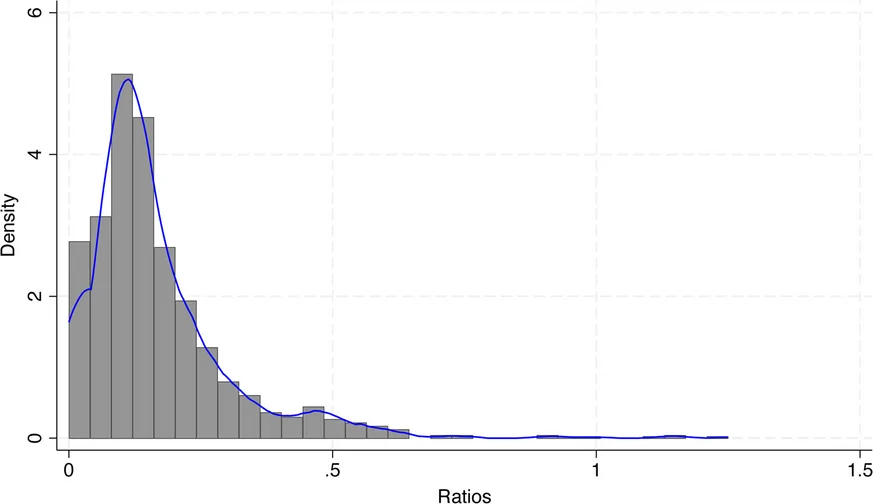
Density of the ratio between NIBRS-reported aggravated assaults with a firearm resulting in an injury and UCR-reported aggravated assaults with a firearm. The histogram shows ratios between NIBRS-reported aggravated assaults with a firearm resulting in an injury and UCR-reported aggravated assaults with a firearm over the period of 2000 to 2020. Observations are at the agency-year level. The overlayed blue line represents a density curve of the ratios

**Fig. 2:**
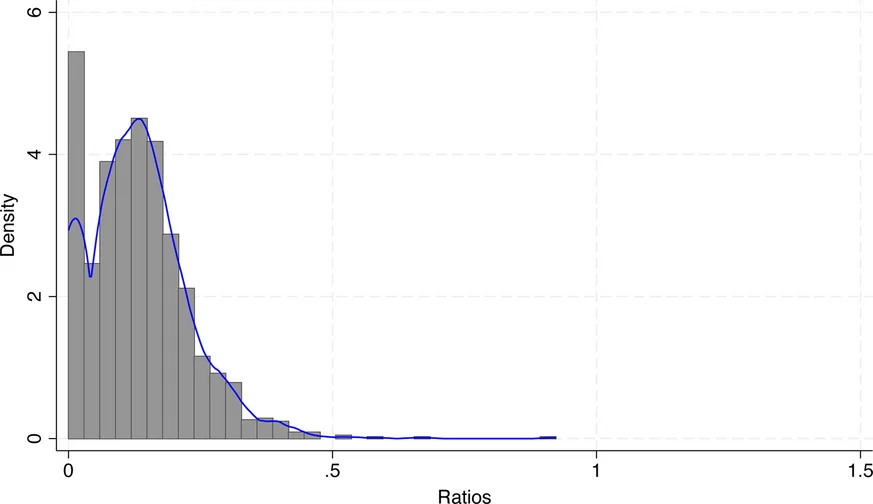
Density of the ratio between NIBRS-reported robberies with a firearm resulting in an injury and UCR-reported robberies with a firearm. The histogram shows ratios between NIBRS-reported robberies with a firearm resulting in an injury and UCR-reported robberies with a firearm over the period of 2000 to 2020. Observations are at the agency-year level. The overlayed blue line represents a density curve of the ratios

According to the UCR, across agencies, an average of 330.03 aggravated assaults (median = 90; SD = 711.64) and 249.16 robberies (median = 67; SD = 520.68) were committed with a firearm each year. According to NIBRS, an average of 45.52 NFS from aggravated assaults (median = 18; SD = 82.61) and 39.13 NFS from robberies (median = 10; SD 81.24) occurred annually. Among agency-year observations, the ratio of NIBRS NFS and UCR gun crimes varied widely for both aggravated assaults (Fig. 1) and robberies (Fig. 2). For each crime category, a cluster of cities exhibited NIBRS NFS-to-UCR gun crime ratios below 0.25. The modes for the ratio of NFS to nonfatal gun crimes were 0.125 for aggravated assaults and 0.00 for robberies. Few observations had a ratio of NFS to crimes > 0.50. Four observations had a ratio of NFS to aggravated assaults with firearms > 1 for aggravated assaults. In total, these ratios suggest that approximately 87% of UCR-reported crime incidents with a firearm had no reported victim that experienced a likely gunshot injury.

We examined first-order differences—the yearly changes—in NIBRS-to-UCR ratios for aggravated assaults and for robberies for each of the 85 agencies (Figs. 4 and 5 of the Appendix). Most agencies had relative year-to-year stability in the ratio of NIBRS NFS to UCR gun crimes. Agencies that experienced the most volatility in these ratios alternated between relatively large increases and decreases across the study period and tended to be smaller jurisdictions with lower volumes of firearm violence, such as Stamford, CT, Lowell, MA, and Fargo, ND. Much greater yearly stability in this ratio was seen in larger cities with more incidents of firearm violence, such as Kansas City, MO, Detroit, MI, and Columbus, OH. Somewhat greater year-to-year volatility in the ratio of NIBRS-to-UCR measures is evident for robberies than for aggravated assaults.

**Fig. 3:**
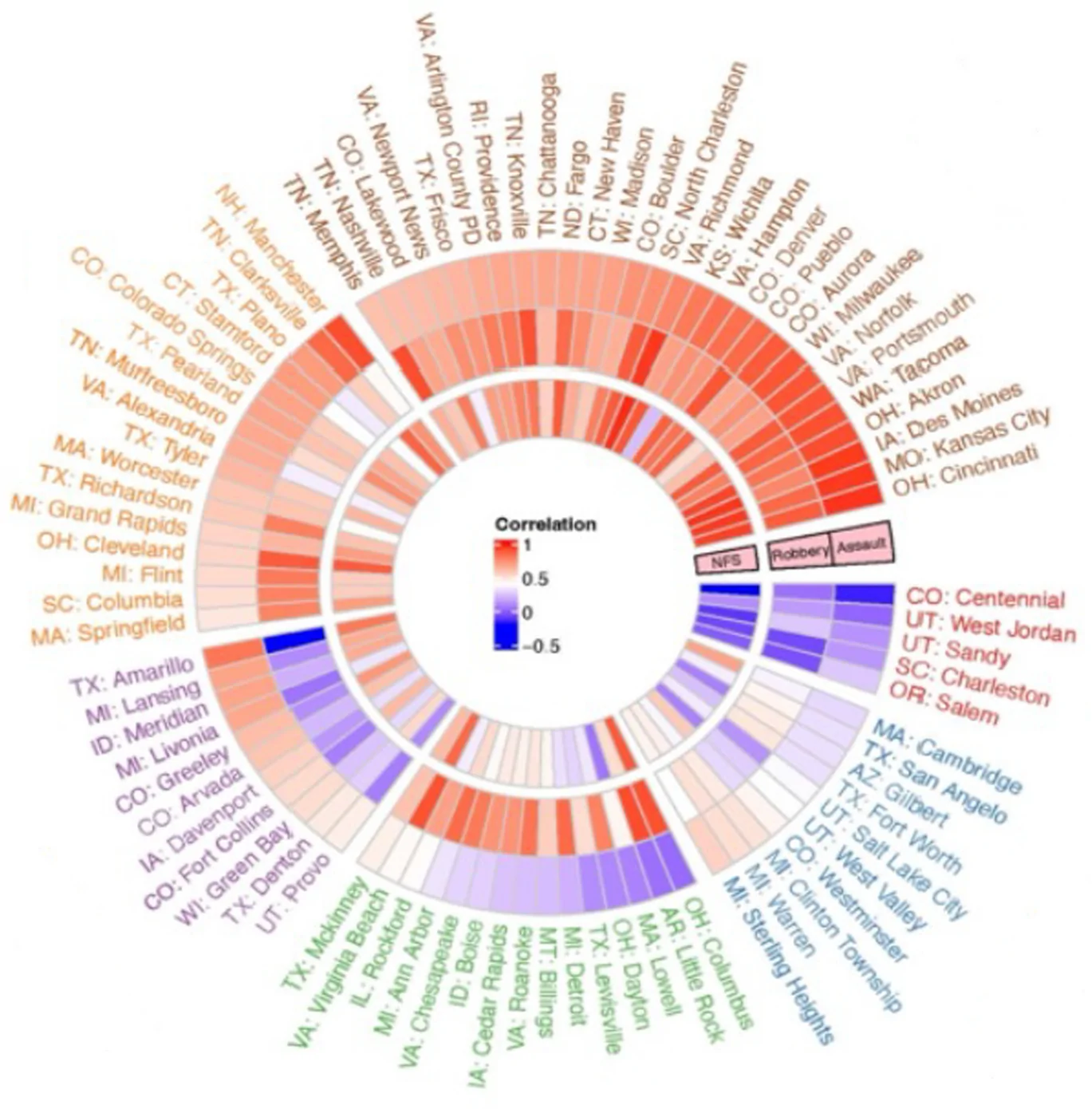
Circos plot of hierarchical cluster analysis of Spearman correlations between annual counts of NIBRS-based nonfatal shootings and UCR-reported nonfatal firearm crimes, 2000–2020

**Fig. 4:**
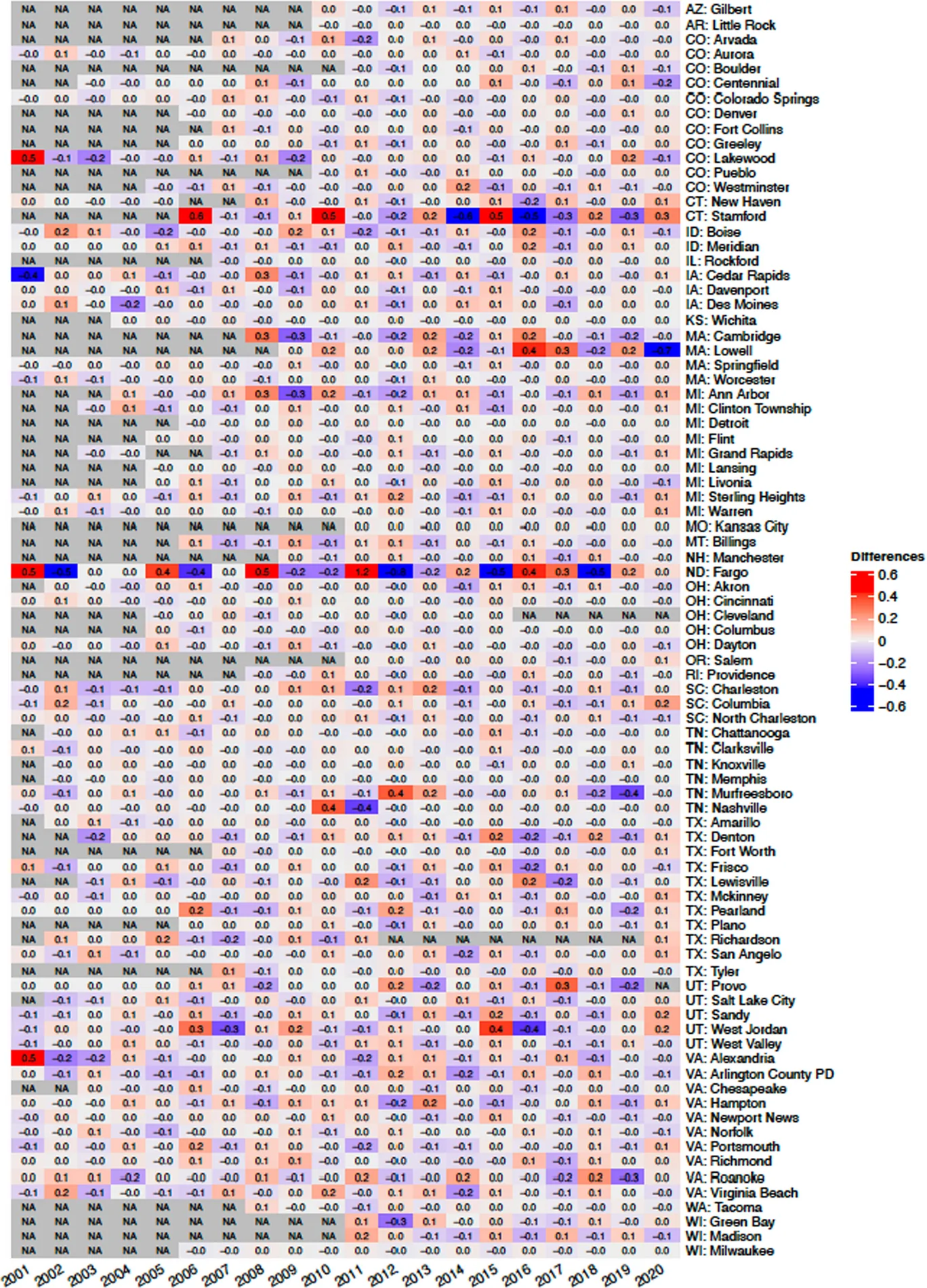
A visual depiction of the annual differences between the ratio of NIBRS reported aggravated assaults with a firearm resulting in an injury and UCR-reported aggravated assaults with a firearm. A value of 0 represents no change in the ratio from the previous year. A value < 0 represents a decrease in the ratio from the previous year. A value > 0 represents an increase in the ratio from the previous year

**Fig. 5:**
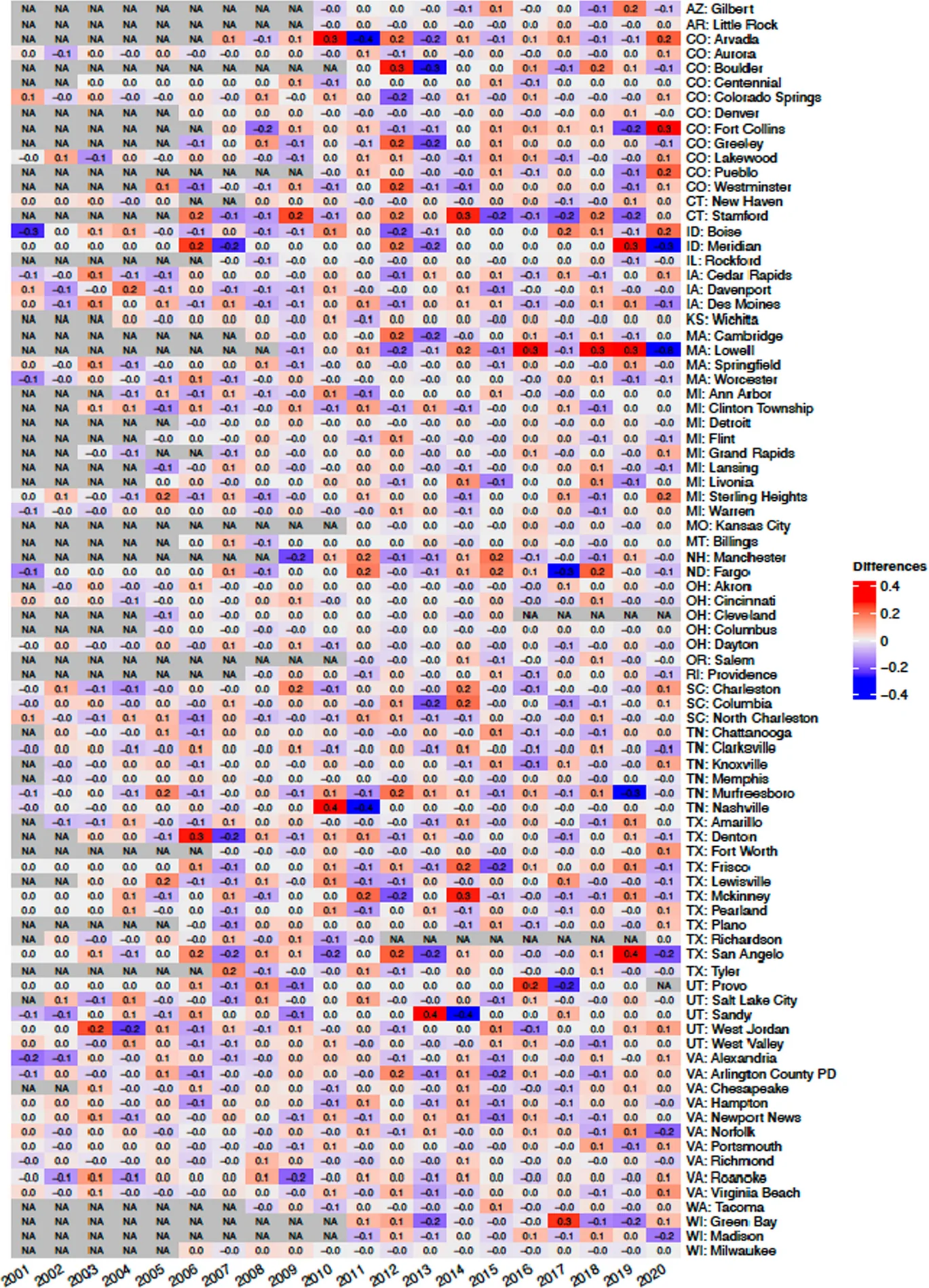
A visual depiction of the annual differences between the ratio of NIBRS reported robberies with a firearm resulting in an injury and UCR-reported robberies with a firearm. A value of 0 represents no change in the ratio from the previous year. A value < 0 represents a decrease in the ratio from the previous year. A value > 0 represents an increase in the ratio from the previous year

Figure 3 charts the hierarchical cluster analysis with a Circos plot of the Spearman ranked-based correlations between annual counts of UCR gun crimes and annual counts of NIBRS NFS for all agencies. This circular layout was segmented into three concentric rings. The outermost two rings correspond to the correlations between UCR-reported criminal incidents and NIBRS nonfatal firearm injuries for aggravated assaults (outer ring) and robberies (middle ring). The innermost ring represents the correlations between the sum of all UCR gun crimes and the sum of NIBRS NFS.

This analysis identified six distinct groups, each seamlessly translating to an arc within the circular arrangement. From the top right and moving counterclockwise, Category 1 comprises 28 agencies (32.9%) that exhibit high positive correlations between NIBRS NFS and the UCR across aggravated assaults and robberies committed with firearms. Category 2 encompasses 16 agencies (18.8%) that display medium positive correlations between NIBRS NFS and UCR measures across aggravated assaults and robberies. Category 3 includes 11 agencies (12.9%) that show medium to high positive NIBRS NFS-to-UCR correlations among aggravated assaults and low positive to negative correlations for robberies. Category 4 comprises 15 agencies (17.6%) that have low positive to negative correlations between NIBRS NFS and the UCR for aggravated assaults, but medium to high correlations between NIBRS NFS and the UCR for robbery. Category 5 encompasses 10 agencies (11.8%) that have low positive correlation between NIBRS NFS and UCR for both aggravated assaults and robberies. Lastly, Category 6 includes 5 agencies (5.9%) characterized by low positive to negative correlations between NIBRS NFS and UCR for both aggravated assaults and robberies.

When we combined sections of the arcs, 55 (64.7%) of the agencies had medium-to-high Spearman correlations between NIBRS NFS and UCR for aggravated assaults, but 30 (35.3%) had low-positive-to-negative correlations between the two data sources. Fifty-nine (69.4%) of the agencies had medium-to-high positive correlations between NIBRS NFS and UCR for robberies, but 26 (30.6%) had low-positive-or-negative correlations between the measures. In sum, 51 (60.0%) of the agencies exhibited low-positive-to-negative correlations between NIBRS NFS and UCR for at least one of the crime categories.

## Discussion

To accurately study and effectively address firearm violence, law enforcement agencies and public health systems need to have accurate and reliable data. Our findings highlight how difficult it is to estimate the incidence and trends of nonfatal gunshot victimization with data from the FBI’s UCR-SRS and NIBRS. According to NIBRS records, approximately 87% of UCR nonfatal gun crimes in this study had no victims who sustained injuries consistent with a gunshot wound. Furthermore, this percentage varied greatly across agencies. Relatively large yearly changes in the ratio of NIBRS NFS to UCR gun crimes were common among agencies that served smaller jurisdictions. Further, researchers must be cautious when using both data systems to compile longitudinal data. Approximately 2% of agency-year observations for NIBRS were incomplete because only a few months of data were reported or injury codes were missing entirely. Smaller agencies may have experienced more difficulties in initial years of reporting to NIBRS because of smaller staffs, lack of resources, or outdated technology [19].

Among the included agencies, more than half (51 of 85) exhibited low positive to negative correlation for aggravated assaults, robberies, or both. This finding highlights the difficulty of extrapolating data on violent crimes that involve a firearm to the incidence and trends of NFS. It also raises several reasons for concern. First, the vast majority of UCR gun crimes do not involve an individual sustaining a gunshot wound. However, there are inherent differences between nonfatal firearm crimes that involve a gunshot wound and those that do not, including motivation or intent related to the incident. Relying on firearm crime data without information on gunshot injury does not accurately depict the extent or impact of NFS. Second, variations in firearm crimes across place and time may not reflect variations in NFS because the reporting practices of local law enforcement agencies may be inconsistent. For example, revised criminal definitions or restrictions in data systems (e.g., the hierarchy rule used by the UCR-SRS) can influence reporting of gun crimes. Lastly, counting firearm crimes does not account for incidents that involve multiple victims. The use of national statistics on firearm crimes is important to the field of violence prevention, but the field must be aware of the inability to draw conclusions on NFS from these data.

The volatility in the ratio of NIBRS NFS to UCR gun crimes within jurisdictions might be explained by fluctuations in small sample sizes of NFS over time, especially among smaller jurisdictions. Alternatively, changes in these ratios in succession may be suggestive of changes in reporting practices. No official definition exists of what constitutes an NFS. Scholars have suggested establishing a clear definition that will assist agencies when reporting and ensure better consistency across and within jurisdictions [7]. We urge the federal government to establish a definition for NFS, as suggested by Dr. Natalie Hipple [7]: “a surviving victim who has experienced a penetrating wound caused by a projectile from a weapon with a powder discharge or explosive as defined by federal law.”

As we observed, most firearm crimes do not result in injury. National Crime Victimization Survey data between 2000 and 2024 indicate that approximately 10% of aggravated assaults committed with firearms and 12% of robberies committed with firearms involved injuries requiring medical treatment [20]. However, just over a quarter of firearm victimizations are reported to law enforcement [21]. NIBRS offers a better metric of NFS than does the UCR-SRS because it includes additional information on sustained injuries. However, NIBRS still provided an inaccurate depiction of the extent of nonfatal firearm violence. Our coding of NFS included severe lacerations, major injuries, or internal injuries. All of these injuries could be sustained by other means during an incident, meaning our study might have overestimated the extent of crime-related NFS. Based on internal Detroit Police Department records of NFS over a 4-year span, approximately 2,000 aggravated assaults with a firearm and injury, as reported to NIBRS, were unmatched to internal records of confirmed NFS [22]. The recent update to NIBRS to include “gunshot wounds” as an injury code should improve upon this historic limitation in federal data systems. To examine the impact of this change, future researchers may find it useful to examine changes in the injury codes endorsed for crimes using a firearm. When using these data, researchers must be aware of these limitations, especially in analyses that combine UCR and NIBRS data to compare trends. To overcome shortcomings in national databases, some law enforcement agencies maintain internal systems to identify and track NFS, independent from data they are federally required to report. Although they provide detailed data on NFS, these internally maintained records are time- and resource-intensive [7]. Most law enforcement agencies design their data management systems to collect only information they are required to report to the federal government [7]. The inclusion of “gunshot wound” as a separate injury category in NIBRS should improve consistency across agencies in capturing detailed data on NFS and reinforces the need to establish a federal definition of NFS.

This study had several limitations to consider. We conducted our analyses among agencies that began reporting data to NIBRS before it was mandated. Therefore, selection bias may occur between agencies that opted into earlier reporting and those that did not. Additionally, our sample included 85 medium- to large-sized cities; thus, our results may not be generalizable to all jurisdictions, especially those that are more rural. Law enforcement data provide a more accurate depiction of nonfatal firearm assaults than do other data sources [5]. However, gaps remain. For criminal incidents to be recorded, typically someone must make a call to emergency services. Then, law enforcement officers must respond to that call, determine that a crime has been committed, and complete the necessary reports after addressing the situation. Notably, findings from the National Criminal Victimization Survey indicate that nearly 40% of respondents who experienced firearm crime victimization did not report the incident to police, often due to fear of reprisal and lack of trust in law enforcement [17, 23]. Consistency in reporting is also likely to differ between periods when firearm violence is trending high and more peaceful times, as political pressures stemming from high rates of violent crimes may create incentives for police to underreport crimes in which victims sustain no serious injuries. Researchers should be cautious about assuming that variations in UCR gun crimes reflect variations in NFS. Because the UCR-SRS institutes a hierarchy rule, which was removed by NIBRS, it is possible that incidents involving an aggravated assault were coded as a more serious crime when reported to UCR-SRS. The UCR-SRS limited the reporting of weapons to aggravated assaults and robberies. Even if a firearm was used in another type of violent crime, it would not be recorded [15]. Prior research has highlighted that this gap contributes to some of the underreporting of firearm-related crimes in studies that use UCR-SRS data [14].

As the field of firearm violence prevention continues to grow, there is a need to ensure the quality and accuracy of data. The FBI’s transition from the UCR to NIBRS will address several gaps in data reporting and provide richer incident-level detail. However, gaps in participation and discrepancies in data reporting across agencies still affect NIBRS data. Our findings highlight inherent limitations of any research that uses historical data from either of the FBI’s data reporting systems to examine NFS. NIBRS including “gunshot wound” as an explicit injury code is a monumental step to advancing firearm violence prevention [7, 22]. Next, establishing a standardized definition of NFS would significantly improve the quality of data available to practitioners, policymakers, and researchers in improving public health and safety.

## Data Availability

The data that support the findings of this study are available from the corresponding author upon reasonable request.

## Appendix

Fig. 4 and Fig. 5
